# A hepatic antimicrobial peptide, hepcidin from Indian major carp, *Catla catla*: molecular identification and functional characterization

**DOI:** 10.1186/s43141-022-00330-7

**Published:** 2022-03-28

**Authors:** P. P. Athira, V. V. Anooja, M. V. Anju, S Neelima, K. Archana, S. Muhammed Musthafa, Swapna P. Antony, I. S. Bright Singh, Rosamma Philip

**Affiliations:** 1 Department of Marine Biology, Microbiology and Biochemistry, School of Marine Sciences, Cochin University of Science and Technology, Fine Arts Avenue, Kochi, Kerala 682016 India; 2 National Centre for Aquatic Animal Health, Cochin University of Science and Technology, Kochi, Kerala 682016 India

**Keywords:** Hepcidin, Antimicrobial peptides, Carp, *Catla catla*

## Abstract

**Background:**

Increase of antibiotic resistance in pathogenic microbes necessitated novel molecules for curing infection. Antimicrobial peptides (AMPs) are the gene-encoded evolutionarily conserved small molecules with therapeutic value. AMPs are considered as an alternative drug for conventional antibiotics. Hepcidin, the cysteine-rich antimicrobial peptide, is an important component in innate immune response. In this study, we identified and characterized hepcidin gene from the fish, *Catla catla* (Indian major carp) and termed it as *Cc*-Hep.

**Results:**

Open reading frame of *Cc*-Hep consists of 261 base pair that encodes 87 amino acids. *Cc*-Hep is synthesized as a prepropeptide consisting of 24 amino acid signal peptide, 36 amino acid propeptide, and 26 amino acid mature peptide. Sequence analysis revealed that *Cc*-Hep shared sequence similarity with hepcidin from *Sorsogona tuberculata*. Phylogenetic analysis indicated that *Cc*-Hep was grouped with HAMP2 family. Structure analysis of mature *Cc-*Hep identified two antiparallel beta sheets stabilized by four disulphide bonds and a random coil. The mature peptide region of *Cc*-Hep has a charge of + 2, isoelectric value 8.23 and molecular weight 2.73 kDa.

**Conclusion:**

Functional characterization predicted antibacterial, antioxidant, and anticancer potential of *Cc*-Hep, which can be explored in aquaculture or human health care.

## Background

The overuse and misuse of antibiotics has stimulated the bacterial evolution towards resistance development, as an adaptive mechanism for survival. To overcome this situation an urgent need for new therapeutic agents are required. Natural products have proven to be rich source of potent compounds having health promoting benefits with specific application in modern medical field [3–5, 10, 13, 26, 29, 34].

Antimicrobial peptides (AMPs) are evolutionarily conserved host defense peptides, distributed widely in nature as an innate immune molecule [32]. AMPs have broad spectrum activity against bacteria, virus, fungi, and parasites. The aquatic environment contains wide variety of pathogens and hence the innate immune system, the first line of defence in fish is highly significant [16, 59, 61]. AMP production was found to enhance in response to infection and exhibit broad spectrum antimicrobial activity against fish and human pathogens [35]. Presence of AMP in fish mucus prevents the colonization of pathogens [51, 60, 62]. Hepcidin, cathelicidin, betadefensin, piscidin, and histone-derived AMPs are the five different classes of AMPs in fishes [35, 50].

Hepcidins are an important group of cationic short peptides with roles in innate immunity and iron homeostasis. Mammalian hepcidins are reported to have both antibacterial as well as iron regulatory mode of action [39, 47, 48, 54]. Hepcidin was first reported in humans and later from other vertebrates [36, 53]. Hepcidin was initially isolated from human urine and was also identified from blood ultra-filtrate which led to naming it as LEAP1 (liver-expressed antimicrobial peptide) [36]. Recently it was renamed to hepcidin due to its hepatic origin and antibacterial activity in vitro [46]. In response to inflammation and iron overload, liver produces hepcidin [24]. Liver synthesizes hepcidin as a prepropeptide and subsequently the signal peptidase and propeptide convertase cleavage results in release of mature peptide [36, 53]. Four intramolecular disulphide bonds formed by eight conserved cysteine residues offer hepcidin a hair pin structure [71]. Number of cysteines can vary from four to eight residues [68]. Fish hepcidin gene contains three exons separated by two introns [25, 30]. Fish hepcidins are categorized into two, HAMP1 and HAMP2 based on amino acid sequence, cationicity, and the iron binding motif DTHFP or QSHLS. HAMP1 hepcidins has similarity with mammalian hepcidins whereas HAMP2 present only in acanthopterygians except *Chlorophthalmus bicornis* [19]. Diversification and duplication of gene resulted in the formation of multiple copies of hepcidin in the genome, identified up to 8 copies [23, 58, 73]. Hepcidin is a versatile molecule proposed to have antimicrobial [14, 17, 22, 38, 40, 42, 75], anticancer [18, 21], antiparasitic [75], and immunomodulatory [65] functions. *Catla catla* (Hamilton, 1822) is the fastest growing species among the Indian major carps and is an important component in polyculture system due to its surface feeding behavior [33]. Higher consumer demand gives *Catla catla* higher economic value. In the present study, an antimicrobial peptide hepcidin *Cc*-Hep was identified from Indian major carp, *Catla catla*. The study mainly focussed on molecular and functional characterization of hepcidin molecule to understand the bioactive potential of this molecule that can be explored for application in aquaculture and medicine.

## Methods

### Sample collection

Live sample of the Indian major carp, *Catla catla*, was collected from Prakrithi fish farm (Kerala, India) and transported to the laboratory in live condition. The fish was killed humanely, blood and gill samples were collected and stored in TRI reagent (Sigma) at – 20 °C until processed.

### Total RNA isolation and reverse transcription

The total RNA was extracted from blood and gill samples in accordance with the manufacturer’s protocol. Quantity of RNA was checked spectrophotometrically at 260 nm and 280 nm. RNA sample with absorbance ratio (A260:A280) greater than 1.8 (good quality RNA) was selected for the current work. The first strand cDNA was synthesized in a 20 μL reaction volume containing 5 μg total RNA, 1× RT buffer, 2 mM dNTP, 2 mM oligo d(T20), 20 U of RNase inhibitor, and 100 U MMLV reverse transcriptase (New England Biolabs, USA). The reaction mixture was incubated at 42 °C for 1 h followed by an inactivation step at 85 °C for 15 min. Beta actin, a housekeeping gene (Forward 5′ATCATGTTCGAGACCTTCAACAC 3′ and Reverse 5′CGATGGTGATGACCTGTCCGTC 3′) was used to test the quality of RNA.

### Hepcidin amplification and cloning

The PCR amplification of hepcidin from cDNA of the fish *Catla catla* was performed using Hepcidin primers (Forward 5′ CGAAGCAGTCAAACCCTCCTAAGATG 3′ and Reverse 5′ GAACCTGCAGCAGACACCACATCCG 3′) [57]. Reaction was carried out in 25 μl total reaction volume containing 1× standard Taq buffer (10 mM Tris-HCl, 50 mM KCl, pH 8.3), 3.5 mM MgCl2, 200 mM dNTPs, 0.4 mM each primer and 1 U Taq DNA polymerase (New England Biolabs, USA). The PCR condition consisted of an initial denaturation at 95 °C for 2 min followed by 35 cycles at 94 °C for 15 s, 60 °C for 30 s, and 72 °C for 30 s and a final extension at 72 °C for 10 min. The amplicons were analysed in 1.5% agarose gel stained with ethidium bromide (0.3 μg/ml) at 90 V for 60 min and visualized using the gel documentation unit (Syngene G Box).

The purified PCR products were ligated into pGEM-T easy clone vector and transformed into competent DH5α *Escherichia coli* cells as per manufacturer’s protocol (pGEM-T Easy TA Cloning Kit, Promega). The cells were cultured in Luria Bertani agar plates containing ampicillin, IPTG, and X-Gal at 37 °C for 24 h, and the recombinant clones were selected by blue white screening. White recombinant colonies screened using vector-specific primers (T7 F and SP6 R) and hepcidin specific primers. For vector specific amplification, 95 °C for 3 min followed by 35 cycles at 94 °C for 15 s, 57 °C for 30 s and 72 °C for 30 s, and a final extension at 72 °C for 10 min program was used. Amplicons were analyzed on 1.5% agarose gels stained with ethidium bromide (0.3 μg/ml) at 90 V for 60 min.

### Plasmid isolation

Recombinant clones were selected for plasmid isolation as per the manufacturer’s protocol using the GenElute HP Plasmid Miniprep Kit (Sigma). Isolated plasmids were analyzed on 0.8% agarose gel. PCR using vector-specific and gene specific primers was done to confirm the presence of insert. The recombinant plasmids were sequenced in ABI Prism 377 DNA sequencer (Applied Biosystem) at SciGenom, India.

### Sequence analysis and molecular characterization

The nucleotide sequence was assembled and analyzed using GeneTool software. The cDNA nucleotide sequence was translated to protein sequence using ExPASy translate tool (http://web.expasy.org/translate/). Homology searches of nucleotide sequence and amino acid sequence were performed using BLASTn and BLASTp algorithm of the National Centre for Biotechnology Information (http://www.ncbi.nlm.nih.gov/blast). The signal peptide region was identified using SignalP 5.0 server (http://www.cbs.dtu.dk/services/SignalP-5.0/). Processing site for propeptidase was determined using ProP 1.0 server (http://www.cbs.dtu.dk/services/ProP). Physicochemical characteristics of the peptide and half-life were analysed using the ProtParam Tool (http://cn.expasy.org/cgi-bin/protparam). Wimley-White whole-residue hydrophobicity and Boman index were predicted using APD3 tool (http://aps.unmc.edu/AP/main.php). Protein motif search was carried out using motif server (https://www.genome.jp/tools/motif) online tool. Coiled coil conformation within the protein was detected using COILS server (https://embnet.vital-it.ch/software/COILS_form.html). Phosphorylation sites in peptide was analysed using NetPhos 3.1 server (http://www.cbs.dtu.dk/services/NetPhos).

To find out the stability of the peptide, cDNA sequence was converted to corresponding RNA sequence using biomodel server (http://biomodel.uah.es/en/lab/cybertory/analysis/trans.htm) and submitted to RNA Fold server program (http://rna.tbi.univie.ac.at/cgi-bin/RNAfold.cgi) to visualize the RNA structure with minimum free energy (MFE). Hydrophobicity of peptide was analysed using the Kyte-Doolittle plot using the ProtScale tool of ExPASy (http://web.expasy.org/protscale).

### Molecular modelling and structure prediction

The secondary structure prediction of *Catla catla* pre-prohepcidin was analysed using PSIPRED (http://bioinf.cs.ucl.ac.uk/psipred/). The tertiary structure of the *Catla catla* pre-propeptide was identified by SWISS-MODEL server, and the PDB data generated for visualization of spatial structure and bonding patterns of the residues using the PyMOL viewer. The stereo chemical quality of the predicted model was evaluated using Ramachandran plot computed with the PROCHECK (http://services.mbi.ucla.edu/PROCHECK/).

### Phylogenetic analysis

Hepcidin sequences were retrieved from NCBI and ClustalW was used for multi-alignment. Phylogenetic tree was constructed using MEGA 7 by Maximum likelihood (ML) method based on the Jones-Taylor-Thornton (JTT) model with complete deletion of gaps and 1000 bootstrap value.

### Mature peptide characterization

Physicochemical properties of mature *Cc*-Hep was predicted using ProtParam Tool (http://cn.expasy.org/cgi-bin/protparam). Expression probability of *Cc*-Hep in different expression systems was evaluated using codon adaptation index value of the peptide (https://www.biologicscorp.com/tools/CAICalculator/#.Xo15x4gzbIU). Quality of the peptide was analysed in peptide ranker (http://distilldeep.ucd.ie/PeptideRanker/). Peptides having rank score greater than 0.7 indicates its bioactive potential.

### Functional characterization

The antimicrobial probability of *Cc*-Hep mature peptide was analysed using CAMP (http://www.camp.bicnirrh.res.in/) database. Antifungal, antiviral, and antiparasitic activities of peptide were predicted using AntiFP (https://webs.iiitd.edu.in/raghava/antifp/), AVPdb (http://crdd.osdd.net/servers/avpdb/), and ParaPep (http://crdd.osdd.net/raghava/parapep/) respectively. Anticancer activity, anti-angiogenicity, and tumor-homing properties of the peptide were evaluated with AntiCP (http://crdd.osdd.net/raghava/anticp/), AntiAngioPred (http://crdd.osdd.net/raghava/antiangiopred/), and TumorHPD (http://crdd.osdd.net/raghava/tumorhpd/peptide.php) servers respectively. Antioxidative, antihypertensive, anti-inflammatory, and anti-tubercular properties were predicted with AnOxPePred (https://services.healthtech.dtu.dk/service.php?AnOxPePred-1.0), AHTpin (http://crdd.osdd.net/raghava/ahtpin/), AIPpred (http://www.thegleelab.org/AIPpred/), and AtbPpred (http://thegleelab.org/AtbPpred) respectively. Hemolytic activity and half-life of the *Cc*-Hep in intestine like environment was analysed in HemoPred (http://codes.bio/hemopred/) and HLP (https://webs.iiitd.edu.in/raghava/hlp/) servers.

## Results

**Fig. 1:**
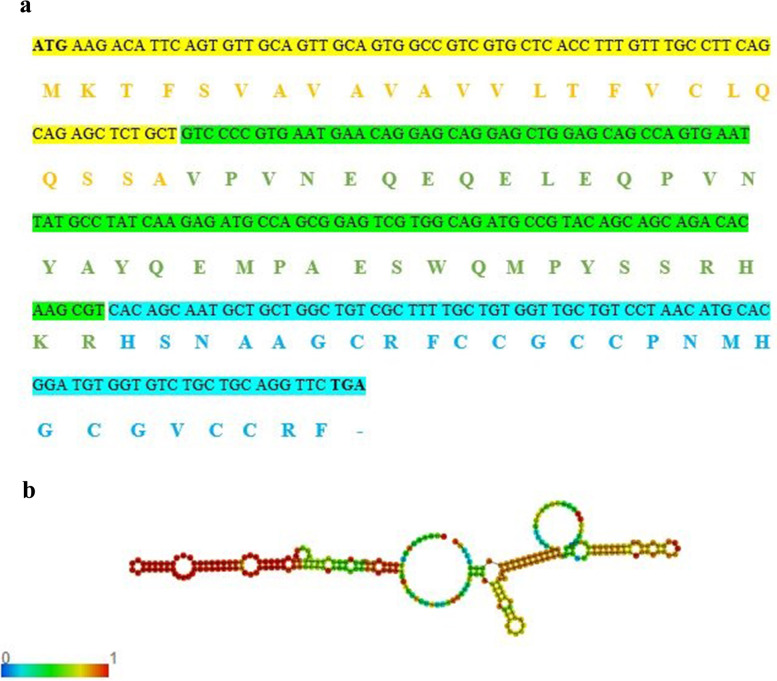
**a** Nucleic acid and deduced amino acid sequences of *Catla catla* hepcidin, *Cc*-Hep (GenBank ID: MW854006). The single letter amino acid code is shown below the corresponding nucleotide sequences. Yellow color specifies signal peptide region, green color propeptide, and blue color mature peptide region. **b** mRNA structure of *Cc*-Hep showing stem loop structure. The mRNA structure colored by base-pairing probabilities. High base-pairing probability is indicated by red color and low base-pair probability indicated by blue color

A 261 bp cDNA fragment encoding 87 amino acids was obtained from the mRNA of *Catla catla* by reverse transcription PCR. Nucleotide and deduced amino acid BLAST analysis identified that the peptide is coming under hepcidin family of antimicrobial peptides. The sequences were deposited in GenBank database under the accession MW854006 (Fig. 1a). Nucleotide similarity search of *Cc*-Hep showed 99% similarity with *Sorsogona tuberculata* hepcidin mRNA (GenBank ID: MN609931.1) and *Leiognathus equulus* hepcidin (Genbank ID: KM034809.1), 87% similarity with *Dicentrarchus labrax* hepcidin 2 (Genbank ID: KJ890400.1), 86% similarity with *Morone chrysops* hepcidin precursor (Genbank ID: AF394246.1), and 82% similarity with *Pagrus major* hepcidin (Genbank ID: AY557619.3).

Protparam analysis predicted the physicochemical properties of *Cc*-Hep, i.e., molecular weight 9.581 kDa, net charge 0.75 and isoelectric point 6.78. *Cc*-Hep was found to be rich in amino acids like valine (11.6%), cysteine (10.5%), alanine (9.3%), and glutamine (8.1%). The aliphatic index and extinction coefficient were identified as 56.63 and 10470 respectively. Half-life estimation of *Cc*-Hep revealed as 30 h in mammalian reticulocytes (in vitro), greater than 20 h in yeast (in vivo) and greater than 10 h in *Escherichia col*i (in vivo). Instability index calculated as 91.42, which classifies the peptide as unstable. *Cc*-Hep is having equal number of positively (arginine + lysine) and negatively charged residues (aspartate + glutamate), highlighting its amphipathicity. Grand average of hydropathicity (GRAVY) was computed as 0.084. Boman index (Protein binding potential) and Wimley-White whole residue hydrophobicity predicted by APD3 server were 1.29 kcal/mol and 15.18 kcal/mol respectively.

Signal 5.0 server predicted the presence of signal peptidase cleavage site in between A^24^-V^25^ producing a signal peptide of 24 amino acids. ProP 1.0 identified the propeptide convertase cleavage site in between R^60^-H^61^, giving rise to 36 amino acid propeptide and 26 amino acid mature peptide with 8 conserved cysteine residues. Pfam identified *Cc*-Hep under Hepcidin family with significant independent *E* value. Serine phosphorylation at positions 5, 49, 55, 56, 62, and one tyrosine phosphorylation site at position 42 were predicted by NetPhos 3.1 server. Phosphorylation in *Cc*-Hep deals with the protease attack protection. No coiled coil formation was detected in *Cc*-Hep.

**Fig. 2:**
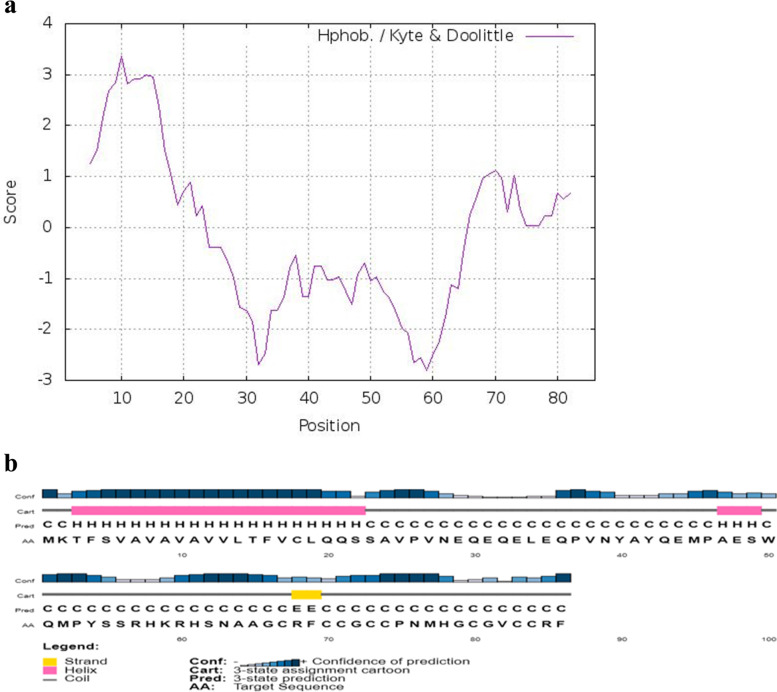
**a** Kyte-Doolittle plot showing hydrophobicity of *Cc*-Hep. The peaks above the score (0.0) indicate the hydrophobicity. The *X* axis is represented by amino acid sequence positions and *Y* axis by hydrophobicity score. **b** Secondary structure of *Cc*-Hep peptide predicted using PSIPRED server. Alpha helical structure represented by pink color, beta strand by yellow color and coils by grey color respectively

Stem loop is a vital component in the structure of RNA. *Cc*-Hep stem loop consists of hairpin loop, internal loop, multi loop, and bulges which gives the structural integrity (Fig. 1b). Minimum free energy (MFE) predicted was − 93.00 kcal/mol indicating that the mRNA is stable and well structured. Hydrophobicity of *Cc*-Hep was analysed by kyte-doolittle plot. Hydrophobic residues are more concentrated in signal peptide region, involved in proper protein translocation (Fig. 2a).

**Fig. 3:**
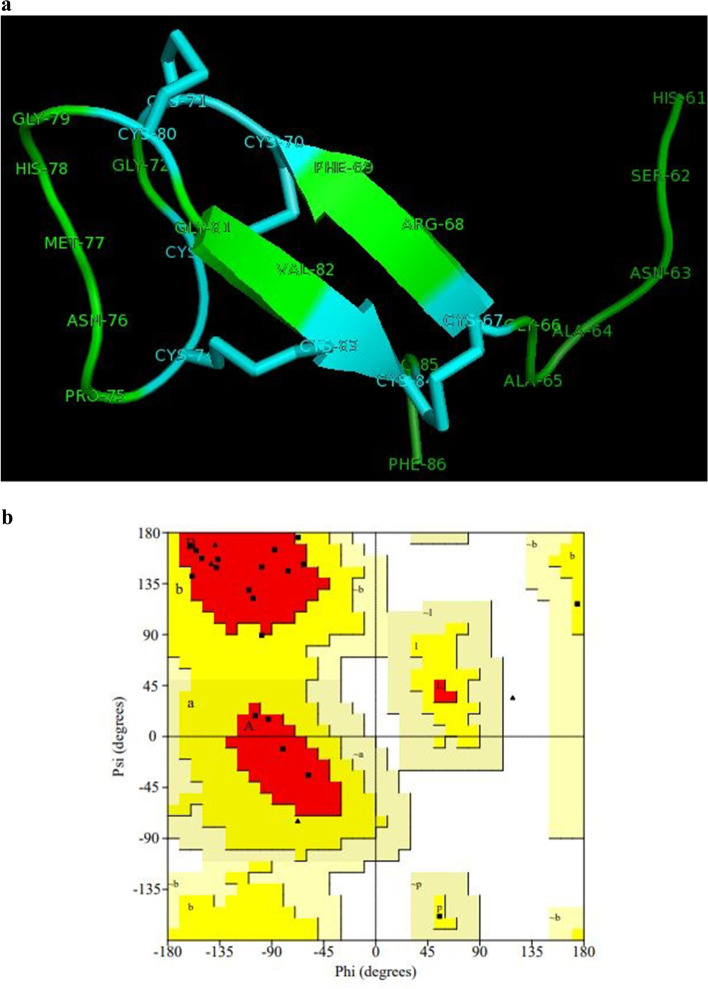
**a** Three dimensional structure of *Cc*-Hep constructed using homology modelling by SWISSMODEL server. Cyan color indicating disulphide bonds. **b** Ramachandran plot for the predicted three dimensional structure of *Cc*-Hep using PROCHECK server

Secondary structure of *Cc*-Hep showed α-helical region followed by beta sheet and random coiled regions. N-terminal α-helical structure formed the signal peptide region, helix and random coils the propeptide, beta sheet and random coiled structure the mature peptide (Fig. 2b). Arginine and phenylalanine of mature peptide formed the beta hairpin structure. The beta hairpin was formed by two beta strands that are adjacent in their primary structure but oriented in an antiparallel direction. Secondary structure is stabilized by 4 disulphide bonds (C^67^-C^84^, C^70^-C^83^, C^71^-C^80^, and C^73^-C^74^). The three dimensional structure of *Cc*-Hep was constructed using solution structure of hepcidin-25 (PDB: 1m4f.1). 3D structure of *Cc*-Hep was found to have two antiparallel beta sheets and random coil strengthened by four disulphide bonds (Fig. 3a). *Cc*-Hep constituted hydrophobic positively charged residues, which permit the effective interaction between peptide and bacterial membrane. Ramachandran plot for the model was constructed with 78.9% residues in most favored regions and 21.1% residues in additional allowed regions. There were no residues in generously allowed regions as well as disallowed regions. A tight clustering of residues at + 120 to + 180° psi value and − 160 to − 45° phi value, clearly validate the antiparallel beta sheet structure of *Cc*-Hep (Fig. 3b).

**Fig. 4:**
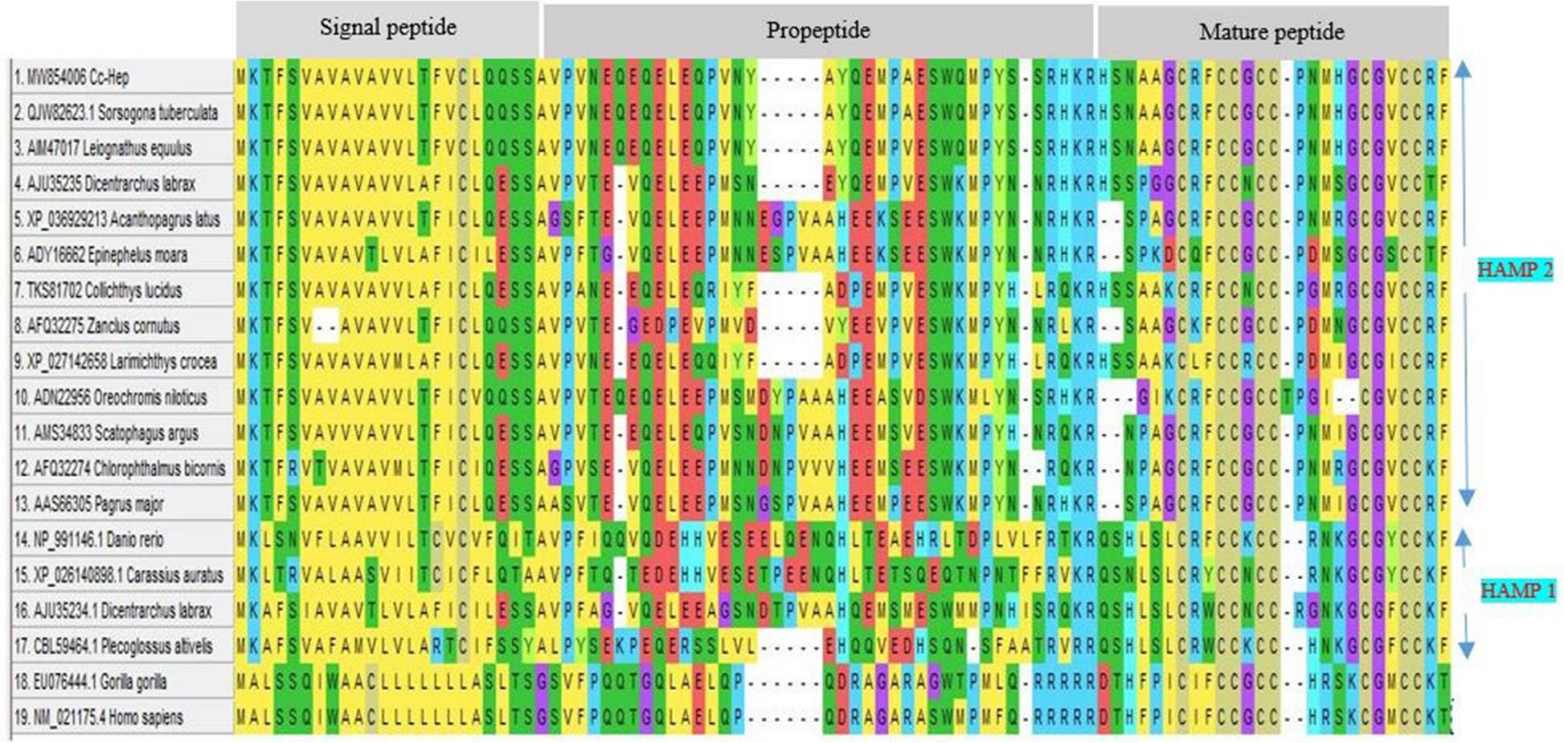
Multiple sequence alignment of *Cc*-Hep using MEGA 6 software. Signal peptide, propeptide and mature peptide of all the sequences are highlighted

**Fig. 5:**
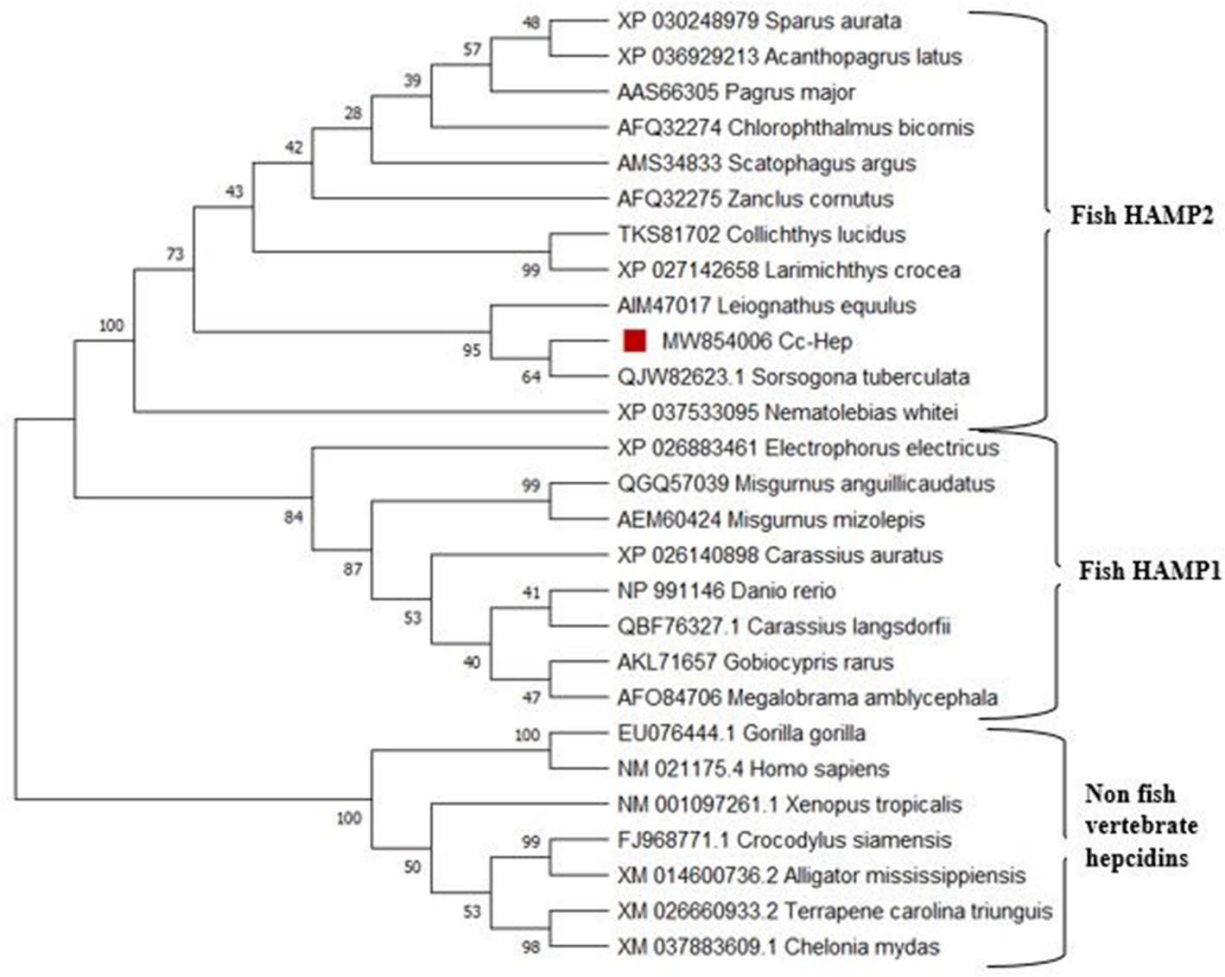
Maximum likelihood tree obtained using MEGA 6 showing the phylogenetic relationship of *Cc*-Hep with other vertebrate hepcidins

The amino acid sequence of *Cc*-Hep aligned with amino acid sequence of previously reported HAMP2 and HAMP1 hepcidins from various organisms (Fig. 4). Signal peptide region was found to be more conserved than propeptide and mature peptide regions. Variation is prominently visible in propeptide part. HAMP1 propeptide showed more variation than HAMP2 propeptide. Negatively charged amino acid, glutamic acid (E) is notable in propeptide part contributing significantly to the anionic nature of pro region. In the signal peptide region, abundance of hydrophobic amino acids like alanine and valine were prominent in fishes, whereas abundance of hydrophobic amino acid leucine was notable in higher organisms. The signature cysteine residues in mature peptide region is associated with disulphide bond formation and proper protein folding. In HAMP1, conserved fish motif QSHL/DTHFP in mammal (first five amino acids of N-terminal mature peptide) was visible. Phylogenetic tree based on the amino acid sequences from different organisms showed two prominent lineages, HAMP1 and HAMP2 (Fig. 5). Non-fish vertebrate hepcidins formed a separate cluster. *Cc*-Hep aligned closely with *Sorsogona tuberculata* hepcidin and deeply nested within HAMP2 clade. Phylogenetic tree confirmed *Cc*-Hep as HAMP2 like peptide and its potential role in antimicrobial activity.

Mature peptide *Cc*-Hep is having a molecular weight 2.72 kDa, charge + 2, isoelectric point 8.23 and 53% hydrophobicity. Mature peptide was found to be rich in amino acids such as cysteine (30%), glycine (15.4%), alanine (7.7%), arginine (7.7%), asparagine (7.7%), histidine (7.7%), and phenylalanine (7.7%). The instability index 35.07, classified the peptide as stable. Aliphatic index and GRAVY of the peptide were 18.85 and 0.342 respectively. The codon adaptation index representing the probable success of heterologous gene expression was predicted to be 0.60 for *Escherichia coli*, 0.69 for *Pichia pastoris*, 0.67 for *Saccharomyces cerevisiae* expression system, 0.85 for *Spodoptera frugiperda* and 0.87 for *Mus musculus*. Peptide rank score was calculated as 0.99 clearly depicting its bioactive potential.

CAMP server confirmed *Cc*-Hep as antimicrobial peptide using Support Vector Machine (SVM) classifier. It was noted that *Cc*-Hep has no antifungal, antiviral, and antiparasitic activity. Server AntiCP classified *Cc*-Hep as an anticancer peptide with 0.81 SVM score. Sequence characterization using both AntiAngioPred and TumorHPD gave significant scores for the antiangiogenic and tumor homing property of the peptide. *Cc*-Hep is an antioxidative peptide with a free radical scavenger score of 0.534. AHTpin, AIPpred and AtbPpred identified its functional types as antihypertensive, anti-inflammatory and antitubercular with prediction scores of 0.53, 0.547, and 0.71 respectively. HemoPred predicted *Cc*-Hep as non-hemolytic peptide, ensuring its safe application. Half-life of *Cc*-Hep was calculated as 0.536 s, indicating its stability in intestine-like environment. All these characteristics mutually compliment, confirming *Cc*-Hep as a potent AMP.

## Discussion

Antimicrobial peptides of fishes represent the first line of defense against infections [43]. Hepcidin, a cysteine-rich antimicrobial peptide is considered as a significant effector molecule in iron regulation and antimicrobial activity in vertebrates [44]. The multiple isoforms of hepcidin present in fish show tissue specific expression pattern [74]. In the present study, a hepcidin AMP *Cc*-Hep was identified from gill RNA of *Catla catla* that shared all the hallmark features of a HAMP2 hepcidin.

Antimicrobial peptides are produced from precursor molecules by hydrolytic cleavage. Generally, hepcidin prepropeptide has 81 to 96 amino acids, with highly conserved signal peptide sequence (24 amino acids), an acidic propeptide (36 to 40 amino acids), and a mature peptide (19 to 27 amino acids). *Cc*-Hep prepropeptide consists of 24 amino acid signal peptides, 36 amino acid propeptides, and 26 amino acid mature peptides. Signal peptide of *Cc*-Hep is found to be conserved and rich in hydrophobic amino acids like valine (25%) and alanine (16.7%). Hydrophobic nature is required for cellular translocation of the peptide. Signal peptidase and propeptidase cleavage is essential for the release of the mature peptide of the prepropeptide. Anionic propeptide deals with the cellular trafficking and charge neutralization of mature peptide region [27, 67]. The RX(K or R) R motif (Furin site) is the processing site for the propeptide convertases in hepcidin [45]. The mature peptide region with a conserved glycine residue and 8 cysteine residues forming 4 intramolecular disulphide bond [11]. *Cc*-Hep mature peptide has a net charge of + 2, isoelectric point of 8.23 and 53% hydrophobicity. The cationicity of *Cc*-Hep mature peptide is mainly due to the amino-terminal portion, which is same for all HAMP2 hepcidins [30].

*Cc*-Hep has all the signature features of hepcidin AMP family and sequence similarity with previously reported hepcidins. Cell membrane destruction is the major way by which AMPs exert its activity, which also explains the reason why AMPs do not produce drug resistance [15, 66]. All the physicochemical parameters of *Cc*-Hep predicted it to be a significant immune molecule. Base pairing ability of mRNA is expressed as its MEF value. Unpaired bases are indicated by positive MEF value, whereas paired bases are indicated by negative MEF value [70]. MEF value of *Cc*-Hep was calculated as − 93.00 kcal/mol indicating the mRNA as mostly paired and only few nucleotides left unpaired.

Structure prediction is important to characterize the function of the protein. The mature peptide of hepcidin has a beta hairpin structure in which the two arms are linked by disulphide bond in ladder-like manner [11]. The mature region of *Cc*-Hep shows two antiparallel beta sheet stabilized by four disulphide bonds (linking the eight cysteine residue) forming a hairpin loop. A notable factor is that the presence of disulphide-bridge between the cysteine residues, near the hairpin turn act as a vital domain in the functioning of hepcidin [25]. Importance of the intramolecular disulphide bond was demonstrated by Hocquellet et al. [31] that synthetic peptide with all cysteines replaced by alanine showed reduced/no antibacterial activity [31]. The disulphide bond has crucial role in the permeabilization of bacterial membrane. *Cc*-Hep also showed spatial separation of hydrophilic and hydrophobic amino acids, a typical feature of membrane disrupting peptide.

Sequence alignment of *Cc*-Hep showed close relationship with previously reported hepcidins, with respect to conserved signal peptide, cysteine configuration and the cleavage site for propeptide convertase. The presence of hydrophobic amino acids like alanine and valine in the signal peptide is notable in fishes whereas leucine is found abundant in higher organisms. Alanine, valine, and leucine are neutral in charge their presence/absence will not affect the total charge of the peptide. HAMP1 conserved motif DTHFP/QSHL deals with ferroportin internalization [46]. Shared ancestry is the reason for sequence similarity. HAMP1 and HAMP2 hepcidin formed separate clusters. *Cc*-Hep occupied the same clade of HAMP2 family of hepcidins. Hepcidins from other vertebrates formed a separate clade. Mammals have single hepcidin gene with both antimicrobial and iron regulation mechanism whereas single copy of HAMP1 gene and multiple copies of HAMP2 genes are present in fishes [30]. Presence of multiple copies of HAMP2 is explained in terms of genome duplication and positive Darwinian selection under different selection pressures [72]. Teleost hepcidins are diverse, mainly due to the diversity of aquatic systems, oxygenation, diversity of pathogens, and different iron concentrations [71]. Bacterial pathogenicity and antimicrobial peptide production is coevolving, by which novel epitope of pathogens produce novel antimicrobial peptides [12, 55].

Oxidation is an important chemical reaction which is present in non-biological and biological processes. Effect of oxidation is the production of free radicals that are unstable and highly reactive causing oxidative stress [41]. Antioxidants are the group of molecules which scavenge and chelate the free radicals. Higher ratio of histidine residue as well as lower ratio of leucine and proline are directly related to antioxidant activity [49]. Peptide *Cc*-Hep was predicted to have antioxidant activity with a notable free radical scavenging score. Antioxidant activity of *Cc*-Hep is by virtue of the higher ratio of histidine and low occurrence of leucine (0%) and proline (3.8%) residues.

Hypertension is one of the major lifestyle associated disease which need new class of drugs with little or no side effects. Occurrence of glycine and phenylalanine is frequent in antihypertensive peptides, whereas amino acids like glutamic acid and aspartic acid occurs rarely. Frequent occurrence of glycine and phenylalanine with rare occurrence of aspartic acid and glutamic acid make *Cc*-Hep a promising lead molecule in antihypertensive therapy [37]. The major bottleneck with the cationic peptide is its short half-life and high susceptibility to degradation by proteases in serum and gut [28]. Size of the amino acid is directly related to the half-life of the peptide, i.e. amino acids like glycine, threonine, alanine and serine (small sized) reduces protease susceptibility and amino acids like phenylalanine, arginine, tyrosine and tryptophan (large sized) reduces the stability of the peptide [64].

Membrane attack is the major mode of action of AMPs. Presence of arginine and leucine increases the haemolytic activity. Presence of glycine is assumed to increase the selectivity towards bacterial membrane and reduces the toxicity to RBC. Absence of arginine and leucine with richness of glycine make *Cc*-Hep a ‘single edged sword’ having both reduced cytotoxicity towards eukaryotic cell and high antimicrobial potential [69].

Natural compounds as anticancer therapeutics has attracted attention because of its cost-effectiveness, nutritional benefits and lesser side effects [6–8]. Anticancer peptides has the ability to discriminate normal and cancerous cell [52]. Hepcidin from tilapia TH2-3 inhibited the growth, proliferation and migration of human fibrosarcoma cell line [21]. TH1–5 peptide specifically lysed cancer cells and strong disorganization of cell membrane was visible under SEM analysis. The peptide also showed antiangiogenicity in HeLa cells [20]. Compounds with anticancer activity display apoptosis and oxidative stress inhibiting the cell proliferation [1, 2, 9]. Server AntiCP identified *Cc*-Hep as anticancer peptide with significant prediction score. AntiAngioPred analysis predicted that the peptide also has antiangiogenic property. Positional preference of amino acids *viz*., cysteine and serine in N-terminal region as well as glycine, cysteine, and arginine in C-terminal region contribute to the antiangiogenicity of *Cc*-Hep peptide. Antiangiogenesis treatments emphasize on principal events like wound healing, migration, extracellular matrix interaction, infiltration, and invasion fuelling tumor growth [1, 2]. The discovery of antiangiogenic property can be explored to inhibit the metastasis of cancer cell [56]. Tumor homing peptides are small peptides, which selectively identify and bind to tumor cells. *Cc*-Hep also showed tumor-homing property. The property was predicted based on SVM model, binary profile model, amino acid composition, and dipeptide composition [63]. Tumor-homing property can be used to deliver drugs at tumor site.

## Conclusion

A HAMP2 family of antimicrobial peptide, *Cc*-Hep was identified and cloned from mRNA transcripts of Indian major carp, *Catla catla.* Analogous nature of *Cc*-Hep to formerly reported hepcidins and its physicochemical properties firmly support its use as an antimicrobial peptide. Functional characterization in silico revealed the peptide for its possible use as antioxidant, antimicrobial and anticancer peptide with minimal side effects. The study also demonstrate the significance of hepcidin antimicrobial peptide in acanthopterygian innate immune system.

## Data Availability

The data generated during and/or analyzed during the current study are not publicly available (exception is GenBank accessions) but are available from the corresponding author on reasonable request.

## Abbreviations

*Cc*-Hep: *Catla catla* hepcidin
HAMP2: Hepcidin antimicrobial peptide 2
HAMP1: Hepcidin antimicrobial peptide 1
AMP: Antimicrobial peptide
